# Early and Delayed Rebound Intracranial Hypertension following Epidural Blood Patch in a Case of Spontaneous Intracranial Hypotension

**DOI:** 10.1155/2022/5637276

**Published:** 2022-03-18

**Authors:** Elham Jafari, Maryam Karaminia, Mansoureh Togha

**Affiliations:** Headache Department, Iranian Center of Neurological Research, Neuroscience Institute, Tehran University of Medical Sciences, Tehran, Iran

## Abstract

**Background:**

Spontaneous intracranial hypotension (SIH) is a secondary headache that has been attributed to a cerebrospinal fluid (CSF) leak. It may resolve spontaneously or require conservative treatment. An epidural blood patch (EBP) with autologous blood is performed in cases exhibiting an inadequate response to conservative methods. Rebound intracranial hypertension (RIH) can develop following an EBP in up to 27% of patients. It is characterized by a change in the headache features and is often accompanied by nausea, blurred vision, and diplopia. Symptoms commonly begin within the first 36 hours, but could develop over days to weeks. It is important to differentiate this rebound phenomenon from unimproved SIH, as the treatment options differ. *Case Presentation*. Here, we present an interesting case of a patient with SIH who was treated with EBP and developed both immediate RIH after 24 hours and delayed RIH 3 weeks following EBP.

**Conclusions:**

Following EBP for treatment of SIH, new onset of headache having a different pattern and location should always be monitored for the occurrence of RIH. A lumbar puncture should be done if the symptoms of elevated CSF pressure become intolerable or if the diagnosis is uncertain. Lack of early diagnosis and treatment and differentiation from SIH can cause complications and could affect the optic nerves.

## 1. Introduction

Spontaneous intracranial hypotension (SIH) is an uncommon but treatable cause of secondary headaches, with an estimated annual incidence of 5 per 100,000 individuals [1, 2]. The most common symptom of SIH is an orthostatic headache which appears shortly after becoming upright and improves or disappears after lying down. The headache is often accompanied by neck pain or stiffness and subjective hearing symptoms [3]. Diagnosis of SIH is primarily made by clinical presentation and can be confirmed by appropriate investigation. The signs in a brain MRI include pachymeningeal gadolinium enhancement, subdural fluid collection, engorged dural venous sinuses, and brain sagging. The use of a dural puncture to measure CSF pressure should be reserved for patients without positive MRI signs [4].

There are several noninvasive treatment options for SIH. These include oral hydration, caffeine, theophylline, and bed rest. An epidural blood patch (EBP) with autologous blood is the most common procedure for spinal CSF leaks when conservative methods prove ineffective. It also functions as a diagnostic and therapeutic tool with response rates that vary from 30% to 90% [5, 6]. If the patient is refractory to multiple EBPs, it is reasonable to explore the leak site by MRI, CT myelography, or cisternography and might lead to consideration of a targeted EBP.

The most common leak location is the thoracic spine, followed by the cervical spine and less commonly the lumbar spine [5, 6]. Without sustained improvement after targeted (to the site of the leak) and/or nontargeted lumbar EBPs, in cases where there is a clear leak, surgical intervention may be required [2, 3].

Rebound intracranial hypertension (RIH) has been reported in 7–27.4% of patients after SIH treatment. This complication has been recognized since 1990 [7]. A headache associated with rebound phenomenon is characterized by changes in headache features that consist of headache that worsens when lying down or is nonorthostatic, is mainly localized in the periorbital area, and is often accompanied by nausea, blurred vision, and diplopia. These symptoms may develop rapidly after EBP or over a period of days or weeks, but they commonly begin within the first 36 hours [8, 9]. It is important to differentiate this rebound phenomenon from unimproved SIH, as the treatment options will differ [2].

Here, we present an interesting case of a patient with SIH treated with EBP who developed both immediate RIH after 24 hours and delayed RIH at 3 weeks following EBP.

## 2. Case Presentation

**Figure 1:**
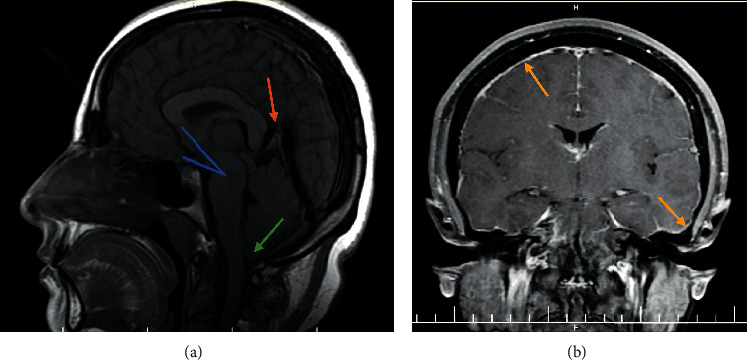
Brain MRI of patient demonstrating (a) cerebellar tonsillar herniation of about 6 mm below the foramen magnum (green arrow), closed pons/midbrain angle (blue lines), and venous engorgement (orange arrow) and (b) mild smooth diffuse dural enhancement (yellow arrows).

A 39-year-old female presented with a history of headache over the previous several months, primarily in the occipital area. The headache was exacerbated by sitting or standing and was relieved by lying down. There was no history of trauma nor underlying connective tissue disorder. She had a normal neurologic exam and a BMI of 25.4. The brain MRI demonstrated mild, smooth, diffuse dural enhancement, cerebellar tonsillar herniation at about 6 mm below the level of the foramen magnum, engorged dural sinuses, and a closed pons/midbrain angle, with the corpus callosum splenium depressing the junction of the inferior cerebral vein and vein of Galen (Figure 1).

She was diagnosed with SIH, and as the symptoms had not responded to conservative treatment with caffeine and bed rest, she was referred for a blind EBP at the lumbar level. The first patch did not produce an adequate response; thus, a second EBP was done after seven days and relieved the symptoms. After several months, she was referred to our center with sudden-onset low back pain radiating to the neck and occipital area. The headache persisted in an orthostatic pattern and was accompanied by nausea and dizziness. She was admitted for further evaluation, and the physical examination revealed no systemic or neurologic deficits. A panspinal MRI was performed which revealed a fluid signal intensity area posterior to the ligamentum flavum at the C2 level without any definite evidence of a defect in the ligamentum flavum. There was no evidence of dural leakage in thoracic region.

Because of the inadequate response to conservative care comprising hydration, bed rest, and caffeine, the patient underwent another EBP. The patch was performed by an expert anesthesiologist with an 18-gauge needle at the L2 and L3 levels using 15 cc of her own blood. The patient maintained a 30-degree Trendelenburg position for 6 hours after the procedure. She experienced immediate relief of the orthostatic headache, but her headache pattern changed by 24 hours following the procedure. It was mostly felt in the frontal area and was improved by sitting, but was aggravated by lying down. Her neurologic examination was normal, and she was diagnosed with early RIH. The headache was relieved after two days of conservative management, and she was discharged from the hospital in good condition.

The patient returned to the hospital after two weeks because of recurrence of the positional headache accompanied by diplopia and blurred vision. The neurologic exam revealed bilateral sixth nerve palsy and swollen optic discs with hemorrhage. The patient was admitted again for neuroimaging and to measure the CSF pressure. The brain MRI and venography showed no abnormalities, and a lumbar puncture was done. The opening CSF pressure was 50 cm H_2_O with normal CSF analysis which confirmed the diagnosis of delayed RIH. She was treated with head elevation and intravenous mannitol, followed by oral acetazolamide, which was titrated up to 500 mg three times a day. The lumbar puncture was repeated after six days, and the CSF pressure was 38 cm H_2_O. Quantitative perimetry showed enlargement of the blind spot, and OCT demonstrated perineural fluid accumulation, but both resolved following treatment for two weeks with acetazolamide. The headache improved within three to four months, and the administration of acetazolamide was tapered-off and discontinued after this period with no recurrence of symptoms.

## 3. Discussion

Spontaneous intracranial hypotension can resolve either spontaneously or with conservative treatment that includes bed rest, oral hydration, and caffeine intake. For patients who do not adequately respond to a conservative approach, injection of autologous blood into the spinal epidural space is the preferred invasive treatment. The success rate for each EBP varies from 30% to 70% for nontargeted patching and up to 87% for targeted patching [2, 5, 6, 9].

The first EBP has been reported to have shown successful clinical improvement in 64% of patients without the need for further intervention. However, a single EBP may not produce a permanent response in some patients, and one or two additional patches at least seven days apart may be required to produce complete relief of symptoms [2, 5, 6, 8, 10, 11]. The mechanism of action of an EBP is not clear, but proposed mechanisms include an increase in intracranial CSF volume and pressure, plugging of the dural leak, and facilitation of rapid healing of the tear. The volumes of blood used in different studies have ranged from small (10 ml) to large (50 ml) amounts [5, 8, 11, 12].

RIH is an infrequently reported complication of EBP in patients with SIH. It results from postprocedural elevation of the CSF pressure. Up to 27% of patients can develop intracranial hypertension following a blood patch for treatment of SIH [1, 6, 13].

The Monro–Kellie hypothesis asserts that the total volume within the skull is constant and volume loss in one compartment will be compensated for by an increase in the other compartments. These compensatory mechanisms in SIH can lead to subdural fluid collection, venous system engorgement, and a possible increase in CSF production. Early RIH that occurs immediately after EBP can be explained by the failure of intracranial venous distension to reverse immediately after repair of the CSF leak. Delayed RIH can develop days to weeks after EBP. The proposed mechanisms for this include upregulation of CSF production and disrupted CSF reabsorption during the period of the CSF leak [6, 14–17].

Our report is an interesting case of a patient with SIH treated with EBP who developed both early RIH after 24 hours and delayed RIH at three weeks post-EBP. A failure to correctly diagnose RIH and its differentiation from SIH can cause complications for a patient because the treatments are different. For a rebound headache, the strategy for management is to lower the CSF pressure [2]. Our patient had a CSF pressure of 50 cm H_2_O, which was immediately treated with head elevation and intravenous mannitol, followed by oral acetazolamide for three to four months.

Kranz et al. [7] reported nine cases of confirmed RIH after EBP; however, most of their patients developed symptoms less than 48 hours post-EBP that included a change in headache location, new nausea, and new blurred vision. The average opening pressure reported was 30 cm H_2_O (range of 22–55), and all cases improved following lumbar puncture and administration of acetazolamide for durations varying from five days to three years. In most RIH cases reported, the onset of symptoms has been rapid (from immediate to three days) and papilledema has been rarely reported, possibly due to the appearance of the usual fundoscopic changes over a period of days to weeks. The CSF opening pressure measurement in these patients ranged from 20 to 55 cm H_2_O [7, 18, 19].

There are few reports of delayed RIH in the literature. In a study of 113 patients with SIH who underwent percutaneous or microsurgical treatment, 27.4% of patients developed RIH. In 23 patients, it occurred within 72 hours, in seven patients in 3–7 days, and in two patients in 7–30 days. Funduscopic examination showed papilledema in only two patients. A lumbar puncture was performed in four patients and found elevated pressure in only one patient (30 cm H_2_O) [19].

CSF pressure is measured infrequently in RIH because of the increased likelihood of postdural puncture headache. Therefore, the diagnosis of rebound intracranial hypertension primarily has been clinical. Lumbar puncture is done when the symptoms of elevated CSF pressure become intolerable, if visual perception problems develop, or if there is doubt about the diagnosis of rebound high-pressure headache.

Our patient developed both types of rebound headache after EBP. The location of the headache changed from the occipital to frontal areas, and the orthostatic feature reversed. The early positional headache was relieved after two days, whereas the delayed one did not respond to conservative management and a lumbar puncture was done after the symptoms and signs of increased intracranial pressure appeared. The opening CSF pressures recorded at the different times were 50 and 38 cm H_2_O.

As in cases like that reported by Kranz et al. [19], our patient responded very well to acetazolamide over a duration of about three months. Patients with RIH generally are reported to be younger, primarily female, and more often showed extradural CSF collection on the spinal imaging. These were all are applicable to our patient.

The discovery of previously elevated CSF pressure in 3% of patients with SIH, as well as the appearance of intracranial hypertension following surgical closure of the CSF leak in some reports, suggest that increased pressure could predispose a patient for a CSF leak at a weak point in the dura. This would be especially true for patients who develop delayed RIH after EBP [20]. Our patient, however, had clinical and imaging presentations of decreased intracranial pressure from the onset of the disease.
